# The Impact of Fiber Source on Digestive Function, Fecal Microbiota, and Immune Response in Adult Dogs

**DOI:** 10.3390/ani14020196

**Published:** 2024-01-07

**Authors:** Miquel Montserrat-Malagarriga, Lorena Castillejos, Anna Salas-Mani, Celina Torre, Susana M. Martín-Orúe

**Affiliations:** 1 Animal Nutrition and Welfare Service, Department of Animal and Food Science, Universitat Autònoma de Barcelona, Cerdanyola del Vallès, 08193 Barcelona, Spain; miquel.montserrat@uab.cat (M.M.-M.); susana.martin@uab.cat (S.M.M.-O.); 2 Affinity Pet Care, Hospitalet de Llobregat, 08902 Barcelona, Spain; asalas@affinity-petcare.com (A.S.-M.); ctorre@affinity-petcare.com (C.T.)

**Keywords:** dietary fiber, dog, microbiota, digestibility, cereal, fruit, short-chain fatty acids, lipid profile

## Abstract

**Simple Summary:**

Dietary fiber has been widely recognized to have a profound impact on gut microbiota and health. However, the ideal fiber intake in dogs still needs to be determined. Fiber is not a single kind of molecule, but a plethora of compounds that are diverse in chemical composition and properties, including solubility, viscosity, and fermentability. Moreover, fiber can be provided in the diet by various fibrous sources that can also offer other bioactive molecules with potential health benefits. In this study, we tested two different diets in dogs, differing in the type of fiber source: derived from cereals or fruits. The results revealed the impacts of both diets on digestive function, lipid metabolism, intestinal fermentation, and fecal microbiota taxonomy, but with a differential footprint. While diets based on cereal fibers appeared to have a greater impact on the microbial ecosystem, diets based on fruit fibers could have additional modulatory properties. This study demonstrated that not only the level or solubility of dietary fiber was important but also the selection of fibrous ingredients in the design of health-promoting diets for dogs.

**Abstract:**

This study evaluated the impact of different fiber sources on intestinal function, fecal microbiota, and overall health in dogs. Twelve dogs were used in a crossover design, involving three periods of 6 weeks and three diets: a low-fiber diet (CTR), a cereal-fiber and beet-pulp-supplemented diet (BRA), and a fruit-fiber-supplemented diet (FRU). Each period included a digestibility trial and fecal and blood sampling in the last week. Short-chain fatty acids (SCFAs) and microbiota taxonomy (16S rRNA Illumina-MiSeq) and functionality (Shotgun-NovaSeq 6000) were determined in the feces. General biochemistry, complete blood cells, and lymphocyte subsets were also analyzed. The fiber-supplemented diets showed lower digestibility without significant changes in the fecal consistency. The BRA diet showed higher total SCFA concentrations (*p* = 0.056), with increases in alpha diversity and particular beneficial genera, such as *Lachnospira*, *Bifidobacterium*, and *Faecalibacterium*. The BRA microbiota was also associated with an overabundance of genes related to carbohydrate and amino acid metabolism. The FRU diet had a distinct impact on the microbiota composition and functionality, leading to higher levels of CD8 lymphocytes. These findings emphasize the importance of selecting the right fiber source when formulating dog diets, as it can have a differential impact on gut microbiota and animal health.

## 1. Introduction

Gut health has been the focus of much research conducted in recent years. The latest advances in the understanding of the role of the intestinal microbiota in various vital functions highlight the relevance of targeting gut health as a driver for the overall well-being of animals [1,2]. Among the different strategies proposed to improve gut health status, supplementation with dietary fiber appears to be an easy and effective approach.

Dietary fiber includes mainly carbohydrate polymers that cannot be digested or absorbed by the small intestine of mammals [3]. Consequently, they can have beneficial effects in the large intestine by selectively stimulating the growth or metabolic activity of microbial species that promote beneficial effects for the host [4]. One of the most commonly reported beneficial effects of dietary fiber in dogs is the increase in fecal short-chain fatty acids (SCFAs), which can play a positive role in intestinal hypertrophy and health [5]. In particular, butyrate, which is one of the main SCFAs in the hindgut, can positively influence the gene expression of the intestinal epithelium and inhibit inflammation and carcinogenesis in humans [6]. Providing a higher amount of fermentable carbohydrates in the diet has also been associated with a decrease in branched-chain fatty acids (BCFAs) and other putrefactive compounds, such as ammonia, aliphatic amines, indoles, and phenols, which are products of amino acid fermentation. In this way, fiber supplementation can promote a shift from putrefactive to saccharolytic fermentation in the hindgut of dogs [7], avoiding potential adverse effects from some putrefactive compounds on colonic health [8].

These changes induced by dietary fiber in gut fermentation are mainly due to changes in the microbiota ecosystem. Fiber supplementation is typically associated with increases in SCFA-producing bacteria from the Firmicutes phylum, such as *Lactobacillus*, *Faecalibacterium*, or *Bifidobacterium*, at the expense of reductions in *Fusobacterium* and some *Clostridium* clusters, which are frequently associated with protein-rich diets in dogs [4,5,9]. Although these changes are generally regarded as positive, the gut ecosystem is highly complex, with variable responses depending on the diet and the host, sometimes making it difficult to predict the precise effects of different fiber sources on microbiota structure and activity.

Moreover, the potential health benefits of dietary fiber in dogs have also been attributed to other effects beyond changes in SCFA production. These effects seen in dogs and other species include changes in the bile acid profile and excretion [10], modulation of the excretion of nitrogen metabolites [11], improvement of the serum lipid profile [12], reduced glucose uptake [13], and enhancement of the immune response [14]. Fiber supplementation has also been used to improve fecal quality in dogs [15] and increase satiety in humans [16], with many of these effects being a result of increased digesta viscosity, delayed gastric emptying, and increased transit time [17].

However, not all types and sources of dietary fiber have the same impact on gut health. There is a wide range of fibers with different chemical and functional properties. Traditionally, different dietary fibers have been classified as soluble or insoluble. Soluble fibers are generally considered fermentable, while insoluble fibers are not. However, it is not as simple as this. In fact, many health properties attributed to different types of fiber do not always depend on their solubility [18]. Moreover, combining different types of fiber can have synergistic effects beyond just complementary modes of action [19]. Therefore, finding an optimal combination of different fiber sources presents a real challenge for the pet food industry.

Various fibrous ingredients have been proposed as potential sources of dietary fiber in dogs. Among them, traditional isolated sources of insoluble and soluble fiber have been fructooligosaccharides and cellulose, respectively. When it comes to food ingredients, beet pulp has been extensively used in the industry due to its desirable ratio of insoluble to soluble fiber (1.9–5.3:1) and its fair amounts of pectins, cellulose, and hemicellulose [20]. In addition to these sources, some alternative fibrous ingredients, like fruits or whole grains, have been introduced more recently. Fruits tend to be rich in pectins, and cereals are rich in inulin. Some ingredients have already been tested in vitro, with apple pomace, carrot pomace, citrus pectins, and pea hulls being highly fermentable ingredients for a dog’s microbiota [21]. In fact, carrot pomace was found to be the most fermented one with the lowest insoluble to soluble ratio (1.9:1), despite having a moderate total dietary fiber content. Citrus pulp and orange fiber are other interesting fiber sources, as they are also highly fermentable. They show high organic matter disappearance and high SCFA production, both in vitro and in vivo in dogs, especially orange fiber, which contains more soluble fiber than citrus pulp [22,23]. Moreover, fruit fibers are rich in bioactive compounds (different kinds of polyphenols) and can have beneficial effects on a dog’s health, aside from their impact on digestive fermentation. For instance, pomegranate peel is rich in ellagitannins, which is a type of polyphenol that has been shown to improve the erythrocytic antioxidant status and serum lipid profile in dogs [24]. In a similar manner, oats are also a good source of bioactive phytochemicals and soluble dietary fiber, especially beta-glucans, which have shown immunomodulatory properties [25] and plasma-lipid-lowering effects in dogs [14]. Despite all this knowledge, there is still a high degree of uncertainty regarding the effects of such alternative sources of fiber on animal health and particularly on the intestinal microbiota when they are introduced into the formulation of commercial extruded dog diets.

Therefore, the aim of this study was to determine the effects of two different combinations of fibrous ingredients on the health of dogs. The initial combination incorporated cereal fibers, such as wheat midds and oat bran, along with beet pulp. The second combination utilized fruit fibers, including citrus pulp, apple fiber, and orange and pomegranate peels, along with cellulose. Their impacts on fecal quality, digestive function, immune system, and intestinal microbiota were assessed. Moreover, the examination of changes in the microbiota was performed based on both the 16S rRNA gene and shotgun sequencing, providing a comprehensive understanding of changes in taxonomy and metagenomic functionality. Possible correlations between changes in the microbiota and fermentation products (SCFAs) were also analyzed in an attempt to better understand the complex underlying mechanisms.

## 2. Materials and Methods

The experimental procedure received prior approval from the Animal Protocol Review Committee of Universitat Autònoma de Barcelona (UAB), following the European Union Guidelines for the ethical care and handling of animals under experimental conditions with Register Number CEEAH: 3010; DMAH: 10166.

A total of 12 healthy neutered adult Beagle dogs (6 males of 14.6 ± 0.80 kg and 6 females of 12.2 ± 0.47 kg, 3–4 years old) were used for this study. The dogs were physically separated into 3 groups of 4 dogs according to their sex and body weight.

### 2.1. Diets

Three experimental dry extruded complete diets were tested in this study (manufactured by Affinity Petcare SA, Hospitalet de Llobregat, Spain): a control diet low in fiber (CTR), the same diet but supplemented with fibrous ingredients from various cereal sources and sugar beet pulp (BRA), and the same control diet but supplemented with fibrous ingredients from various fruit sources (FRU). The fibrous ingredients included in each supplemented diet can be found in Table 1 and the ingredient list of the CTR diet in Table A1. Both fibrous diets also included 0.5% fructo-oligosaccharides as a purified source of soluble fiber, and the FRU diet also had cellulose pellets (2.5%) as a purified source of insoluble fiber. Sugar beet pulp and cellulose pellets were used as sources of soluble and insoluble fiber to ensure a consistent level of total dietary fiber and an equivalent insoluble/soluble ratio. The analyzed chemical composition of the three diets is presented in Table 2. The daily ration was offered individually once a day in the morning. The initial quantity of provided food was determined based on individual metabolizable energy requirement (MER) values, calculated as MER = Coefficient × Body Weight^0.75^, as estimated from prior records for each dog. The average initial coefficient was 117 ± 4.9. Subsequent adjustments to the daily ration were made every two weeks, as necessary, to ensure the maintenance of body weight. These empirical adjustments varied between 5% and 10% in all instances.

### 2.2. Experimental Design

The diets were tested in a crossover design with three periods of 6 weeks each (12 replicates per treatment). Each period consisted of a first transition week during which the animals were gradually introduced to the new diet (over 6 days), followed by four weeks of adaptation and a sixth week for sampling.

During the sampling week, total feces were collected twice a day for 6 days to assess the diet’s digestibility. Before and after the collection of feces, a color marker (FeO_3_) was added to the food to ensure that only the feces belonging to the digestibility trial were collected. Also, during the sampling week, small sub-samples of fresh feces were collected and divided into two aliquots for microbiota and SCFA determination (2 g and 8 g, respectively, from the same stool). All samples were stored at −20 °C until analysis (between 1 and 3 months).

The fecal score was assessed multiple times on a daily basis during the sampling week. Feces were graded on a scale of 1–7 (Fecal Scoring System, Nestle Purina Petcare), with grade 1 representing dry, crumbly feces, and grade 7 representing diarrhea. An average value was calculated for each animal and diet (per period).

Fasting blood samples were taken at the end of each period. Blood was collected using non-heparinized tubes, which were centrifuged at 1500× *g* for 10 min after 30 min, and the serum was collected and frozen at −20 °C until analysis. Additional fresh blood samples were collected with EDTA tubes for complete blood count (CBC) and lymphocyte subset determinations.

### 2.3. Analytical Methods

Collected feces were thawed and dried in an oven at 60 °C until a constant weight was achieved (3 days) to determine the stool dry matter. After drying, the feces collected for each animal during the 6-day collection period were ground and mixed, and a representative sample was taken for further chemical analysis.

All analyses were performed following the AOAC methods. Feed and feces were analyzed for moisture, ash, crude protein, crude fat, NDF (neutral detergent fiber), ADF (acid detergent fiber), and ADL (acid detergent lignin) following the standard procedures of the Association of Official Agricultural Chemists [26]. The specific AOAC methods used for total dietary fiber, soluble dietary fiber, and insoluble dietary fiber were AOAC 2009.01, AOAC 993.19, and AOAC 993.19, respectively. The total polyphenol content was analyzed with the Folin–Ciocalteu reagent, with spectrophotometry at 765 nm [27]. Gross energy was measured using an adiabatic calorimetry bomb. Additionally, purine bases as a microbial marker were analyzed using high-performance liquid chromatography, following the method described by Makkar and Becker [28].

Short-chain fatty acids were analyzed using gas chromatography after an acid–base treatment of the samples, followed by diethyl ether extraction and derivatization with the *N*-(tert-butyl-dimethylsilyl)-*N*-methyltrifluoroacetamide plus 1% tert-butyl-dimethylchlorosilane agent, using the method of Richardson, Calder, Stewart, and Smith [29], which was subsequently modified by Jensen, Cox, and Jensen [30].

Urea, total cholesterol, HDL-c, LDL-c, triglycerides, and glucose were determined in blood serum using photometric tests in an Olympus AU 400 Chemistry Analyzer (Beckman Coulter™, Irving, TX, USA) with Olympus System Reagents^®^ (Antrim, UK).

For the CBC, flow cytometry analysis was performed in a Sysmex™ XN-1000 (Kobe, Japan). For the lymphocyte subset determination (CD4+ and CD8+), immunophenotyping was carried out using a BD FACSCanto™ II Flow Cytometer (San Jose, CA, USA).

DNA extraction from the fresh feces was performed using the QIAamp DNA Stool Mini Kit by Qiagen™ (Hilden, Germany). The quality of DNA extracts was evaluated using spectrophotometry (Nanodrop™, Wilmington, DE, USA) and fluorimetry (Qubit™ HS, Wilmington, DE, USA).

The fecal microbiota taxonomy was determined by sequencing the 16S rRNA V3-V4 regions using the Illumina MiSeq platform. Prior to sequencing, the 16S rRNA gene was amplified using universal primers [31], a universal linker sequence, and indexes and sequencing primers provided by the Nextera XT Index kit (ILLUMINA, San Diego, CA, USA). Raw sequences, namely, forward (R1) and reverse (R2), were imported into the QIIME2 platform [32]. Then, DADA2 plugin [33] was used to denoise and filter the reads. The taxonomy of the resulting ASVs was annotated using ‘blastn’ v2.2.29+ [34] against a 16S-specific database from the NCBI (v. May 2022).

Shannon, Simpson, and richness indexes were calculated using the ’vegan’ R package [35].

A Bray–Curtis dissimilarity matrix and ‘Permutational Multivariate Analysis of Variance’ (PermANOVA) were also performed using the ‘vegan’ R package.

Microbiota functional analysis was approached via metagenome sequencing of the same DNA extracts using the Illumina NovaSeq 6000 platform in a 150 × 2 paired-end configuration. The quality of the resulting sequences was filtered using the BBMap v38.36 program [36], and the canine and human genomes (GCF_000002285 and GRCh37) were eliminated using NCBI databases. ‘SPAdes’ v3.13.0 [37] was used for the reads assembly. The optimal kmer size for the assembly was 127 nt, and contigs less than 500 nucleotides long were filtered.

Open reading frames were predicted with the ‘Prodigal’ v2.6.3 [38] and grouped using the CD-HIT v.4.7 [39] programs. Finally, the functional assignation of predicted genes was done with the ‘ggnog-mapper’ v2.1.5 program [40].

### 2.4. Calculations

The apparent digestibility values were calculated using the following formula:

$$Digestibility=\frac{Ingested-Excreted}{Ingested}\times 100$$

Corrected crude protein (CCP) digestibility was also calculated by subtracting the fecal bacterial nitrogen. Fecal bacterial nitrogen (FBN, in g N/g feces) was estimated using the purine content of feces (analyzed as guanine (G) and adenine (A) in mmol/g feces). Based on previous data, it was estimated that there were 1.5 mmol of purine bases per gram of bacterial N. The formula used was as follows:

$$FBN=\frac{G+A}{1.5}$$

And in this way, the corrected crude protein digestibility was calculated using

$$CCP\;Digestibility=\frac{Ingested-\left(Excreted-MicrobialN\right)}{Ingested}\times 100$$

The hemicellulose (HC) and cellulose (CE) (for digestibility analyses) were calculated from the Van Soest fiber analysis (NDF, ADF, and ADL) with the following formulas:

CE=ADF−ADLHC=NDF−ADF

The metabolizable energy (ME) content of the diets was estimated from the experimental values of digestible energy (DE) and digestible protein (DP) using the FEDIAF Nutritional Guidelines formula [41]:

$$ME=\frac{DE-\left(DP\times 1.25\right)}{Food\;consumed\;(g)}$$

### 2.5. Statistical Analysis

Statistical analyses for performance, fecal quality, alpha diversity, digestibility of nutrients, SCFAs, and blood parameters were conducted using R software, version 4.0.5 for Windows. The data were analyzed with RStudio version 1.4.1106 using analysis of variance (ANOVA) with the general linear mixed model (GLMM) procedure with the ‘nlme’ package, version 3.1-153. Normality and equal variances were checked beforehand in all continuous variables categorized by dietary treatment using the Shapiro–Wilk test. The general model included the diet, period, animal group, and interaction of diet and period as fixed effects and the subject as a random effect.

For taxonomical and genomic differences between the groups, the ‘limma-voom’ R package v.48.3 [42] was used, introducing sequence data without normalization and considering the subject as an effect. To study correlations between microbial abundances and SCFA content parameters, the ‘MaAslin2′ package [43] was used.

Data were presented as least square means ± standard deviation. The level of significance was α = 0.05 (α < 0.1 for trends).

## 3. Results

No relevant incidents were reported during the experiment, except in one period when it was not possible to collect fresh fecal samples from one animal for SCFAs and microbiota analysis. There were no records of any food refusals throughout the study, and the diets were consistently consumed in their entirety by the dogs. The average energy intakes for the CTR, BRA, and FRU diets were 1008 ± 146.5, 1000 ± 123.7, and 980 ± 118.8 kcal ME/day, respectively. The live weights of the animals exhibited slight variations between periods but did not show significant differences between diets or any interactions.

### 3.1. Fecal Quality

Various parameters related to fecal quality are presented in Table 3. The fecal dry matter was lower in the BRA and FRU treatments, which notably increased the daily fecal mass but did not result in significant changes in fecal score. The BRA diet exhibited a higher concentration of fecal bacterial nitrogen than the FRU diet (estimated from the fecal concentration of purine bases).

### 3.2. Apparent Digestibility of Nutrients and Metabolizable Energy

The digestibility of dry matter, organic matter, and gross energy was lower for the BRA and FRU diets compared with the CTR diet (Table 4). In terms of protein, the apparent digestibility was lower with the BRA and FRU diets compared with the CTR diet, with the FRU diet showing the lowest values. However, when the apparent digestibility values were corrected for microbial nitrogen (estimated by purine bases), the BRA diet showed no differences from the CTR diet. In contrast, in terms of fat digestibility, only the BRA diet showed differences from the CTR diet, exhibiting lower values. Regarding fiber, the hemicellulose digestibility was lower with both fibrous diets compared with the CTR diet, but only the FRU diet showed lower cellulose digestibility. Levels of metabolizable energy (ME) estimated from FEDIAF equations were very similar between the fibrous diets but lower compared with the CTR diet.

### 3.3. Fecal Short-Chain Fatty Acids

Table 5 displays the fecal concentration of SCFAs with the different experimental diets in terms of mmol/kg fresh matter and molar proportion. Both fibrous diets showed numerically higher concentrations of total SCFAs compared with the CTR diet, with BRA showing a statistical trend (*p* = 0.057). Significantly higher levels of acetate concentration were also recorded with BRA, and butyrate exhibited a trend to be higher with FRU when compared with CTR (*p* = 0.087). Additionally, BRA and FRU diets were associated with lower concentrations of BCFA. In most of the animals, lactic acid was below the minimum level of detection (1.7 μmol/g feces). In terms of the molar ratio, both supplemented diets were linked to significantly higher percentages of acetate and lower percentages of butyrate and BCFAs.

### 3.4. Serum Biochemistry and Complete Blood Counts

The lipid profile and other biochemical parameters in serum are presented in Table 6. Although no significant differences in HDL and LDL cholesterol were observed, total cholesterol showed a tendency for lower values with the FRU diet compared with the CTR diet. Triglycerides showed significantly lower values with the BRA diet compared with the CTR diet. The glucose levels were not affected by the diets. Urea was significantly lower with the FRU diet compared with the CTR diet, with the BRA diet showing intermediate levels.

Complete blood cell (CBC) counts and lymphocyte subset counts can be seen in Table 7 and were not different between diets. However, some differences were seen in terms of the leukocyte formula; the percentage of lymphocytes showed higher values with the FRU diet compared with the BRA diet. Finally, regarding lymphocyte subsets, the FRU diet showed higher CD8 counts compared with the BRA diet.

### 3.5. Fecal Microbiota Structure and Biodiversity

**Figure 1:**
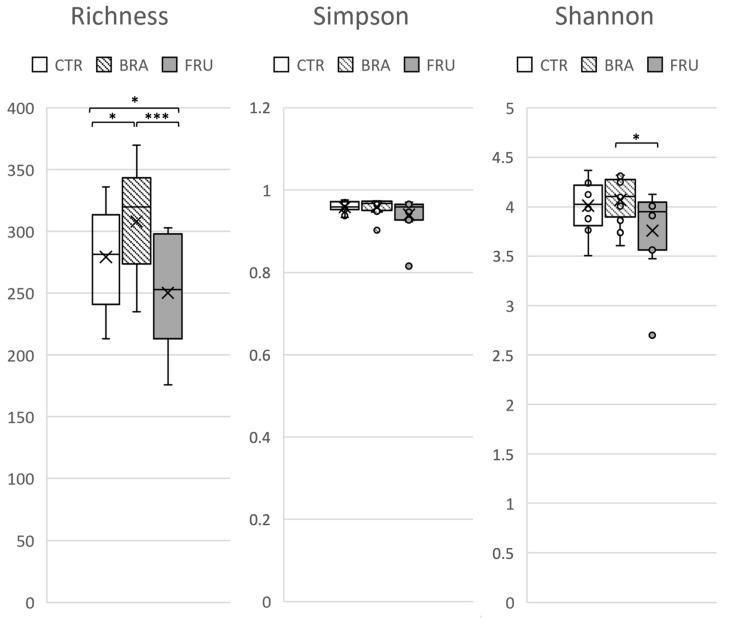
Richness, Simpson, and Shannon indexes for alpha diversity, based on ASVs of dog’s fecal microbiota. CTR—control diet low in TDF (5.7%); BRA—diet enriched with fiber from cereals (12.0% TDF); FRU—diets enriched with fiber from fruits (12.2% TDF). Outliers, the values that are above or below 1.5 times the interquartile range, are represented as circles and the means are represented as X. The central line represents the median. * *p* < 0.05, *** *p* < 0.001.

An average of 74,264 ± 12,661 16S rRNA gene V3-V4 region sequences per sample were obtained, reaching the rarefaction curves’ plateau phase. The sequences were assigned to amplicon sequence variants (ASVs) based on a 97% sequence similarity, obtaining an average of 288 ± 48.7 ASVs per sample. Richness, Simpson, and Shannon indexes were calculated for the alpha diversity assessment and can be seen in Figure 1. Significant differences were observed between treatments in terms of the ASV alpha diversity for the richness and Shannon indexes. The FRU diet showed the lowest richness and Shannon values, and the BRA diet exhibited higher richness values than the CTR.

**Figure 2:**
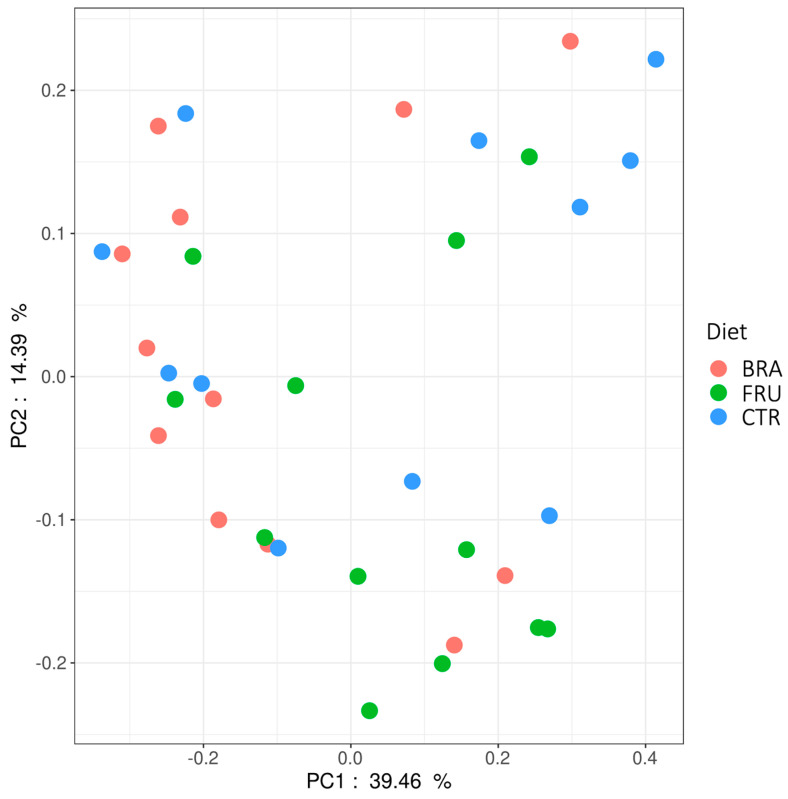
Bray–Curtis principal coordinate analysis plot of sequence data from dog’s fecal microbiota. CTR—control diet low in TDF (5.7%); BRA—diet enriched with fiber from cereals (12.0% TDF); FRU—diets enriched with fiber from fruits (12.2% TDF).

Beta diversity was assessed with a principal coordinates analysis (PCoA) plot using Bray–Curtis distances (Figure 2). A PermANOVA analysis was also performed using the distances, identifying the diet and the animal as two significant factors (*p* = 0.004 and *p* = 0.005, respectively). The animal factor explained 40.5% of the variability, while the diet explained 12.3% of the variability. The plot, however, does not show any clear clustering depending on the diet.

**Figure A1:**
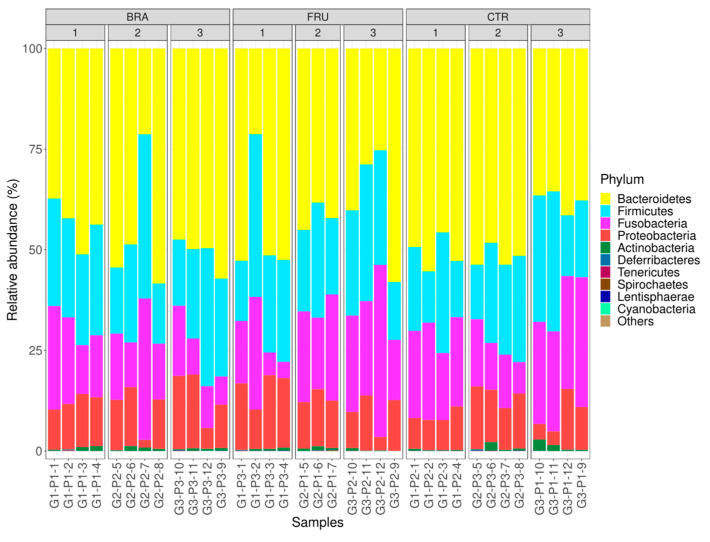
Relative phylum abundance of fecal microbiota in 12 beagle dogs receiving 3 experimental diets differing in their fiber content along three experimental periods (crossover design). Results are shown for each animal in each treatment and period. CTR: control diet low in TDF (5.7%); BRA: diet enriched with fiber from cereals (12.0% TDF); FRU: diet enriched with fiber from fruits (12.2% TDF).

The most abundant phyla were *Bacteroidetes* (45.1 ± 9.97%), *Firmicutes* (23.9 ± 7.49%), and *Fusobacteria* (19.0 ± 8.7%), with non-significant differences between treatments (Figure A1). Differences at the family level between treatments can be found in Table A2. *Prevotellaceae*, *Fusobacteriaceae*, and *Bacteroidaceae* were the most abundant families, but only *Selenomonadaceae* and *Bifidobacteriaceae* showed significant differences between diets. The former was higher with the FRU diet, and the latter with the BRA diet compared with the CTR.

**Figure 3:**
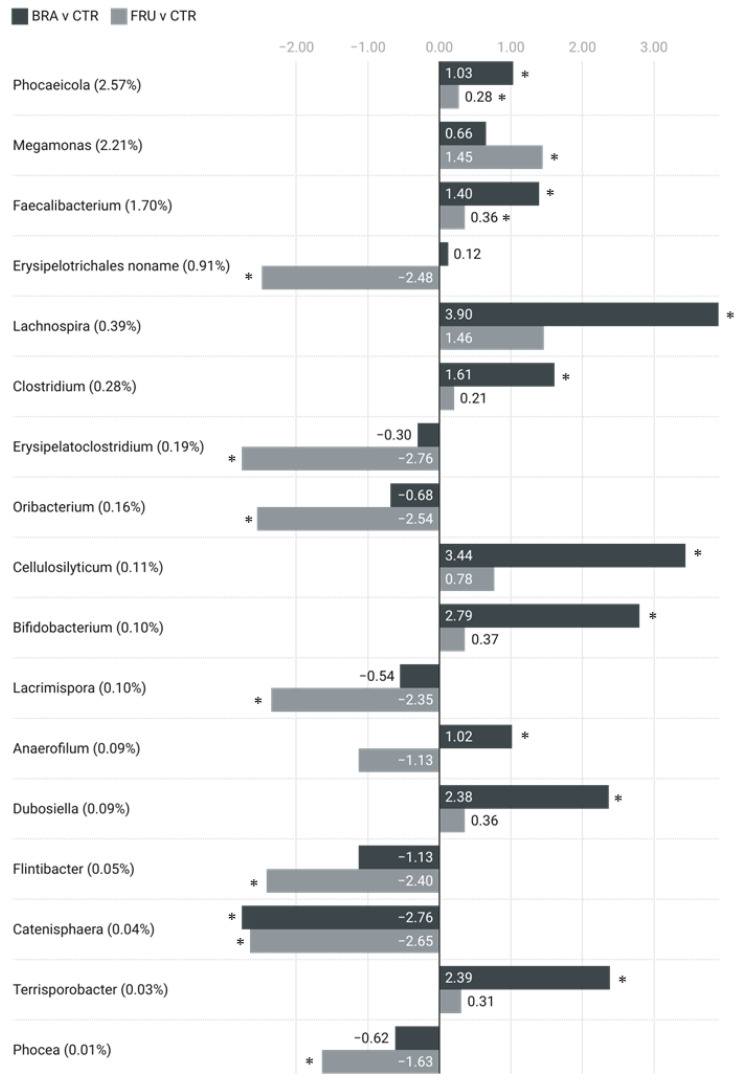
Fecal genera with significant abundance differences between fiber-supplemented diets and CTR. Data presented as logarithmic ASV counts change. Each genus’s overall relative abundance is shown in brackets. Genera are ordered by abundance. CTR—control diet low in TDF (5.7%); BRA—diet enriched with fiber from cereals (12.0% TDF); FRU—diets enriched with fiber from fruits (12.2% TDF). * *p* < 0.05.

When comparing abundances between treatment groups at a genus level, the BRA diet was associated with higher abundances of *Lachnospira*, *Cellulosilyticum*, *Bifidobacterium*, *Clostridium*, *Fecalibacterium*, and *Phocaeicola*, among others, and a lower abundance of *Catenisphaera* compared with the CTR diet. Regarding the FRU diet, it was associated with higher levels of *Megamonas*, as well as *Fecalibacterium* and *Phocaeicola*, but to a lesser degree than the BRA diet. The FRU diet was also associated with lower abundances of *Erysipelatoclostridium*, *Oribacterium*, *Lacrimispora*, *Flintibacter*, and *Catenisphaera* when compared with the CTR diet (Figure 3).

**Figure 4:**
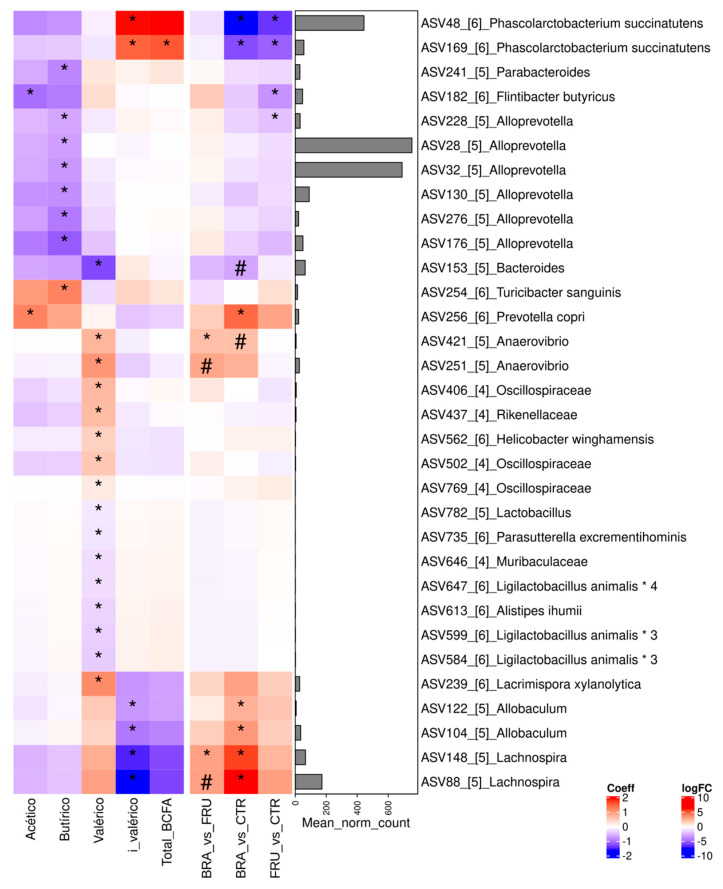
Heatmap of the correlations between SCFAs and specific ASV on the left side of the figure, and log 2 fold-change abundance differences between diets on the right side. Red on the left side means a positive correlation and blue means a negative. On the right side, red means an overabundance of the specific ASV in the former diet in comparison with the other, and blue means an underabundance. Bar graph on the right shows the average normalized counts for each specific ASV. CTR: control diet low in TDF (5.7%); BRA: diet enriched with fiber from cereals (12.0% TDF); FRU: diets enriched with fiber from fruits (12.2% TDF). * *p* < 0.05, # *p* < 0.1.

In an attempt to find possible associations between changes in fermentative activity and microbial groups, correlation analyses were performed between SCFAs and microbial abundances at the ASV level (Figure 4). Butyrate was negatively associated with several *Alloprevotella* ASVs and positively correlated with *Turicibacter sanguinis*; however, none of these microbial groups showed significant changes with the experimental diet. Isovaleric acid showed a strong negative correlation with *Lachnospira* and *Allobaculum* ASVs, which were more abundant in the BRA diet compared with the CTR. BCFAs, particularly isovaleric acid, were positively associated with *Phascolarctobacterium succinatutens*, which was significantly less abundant as a result of the fiber-supplemented diets.

**Figure 5:**
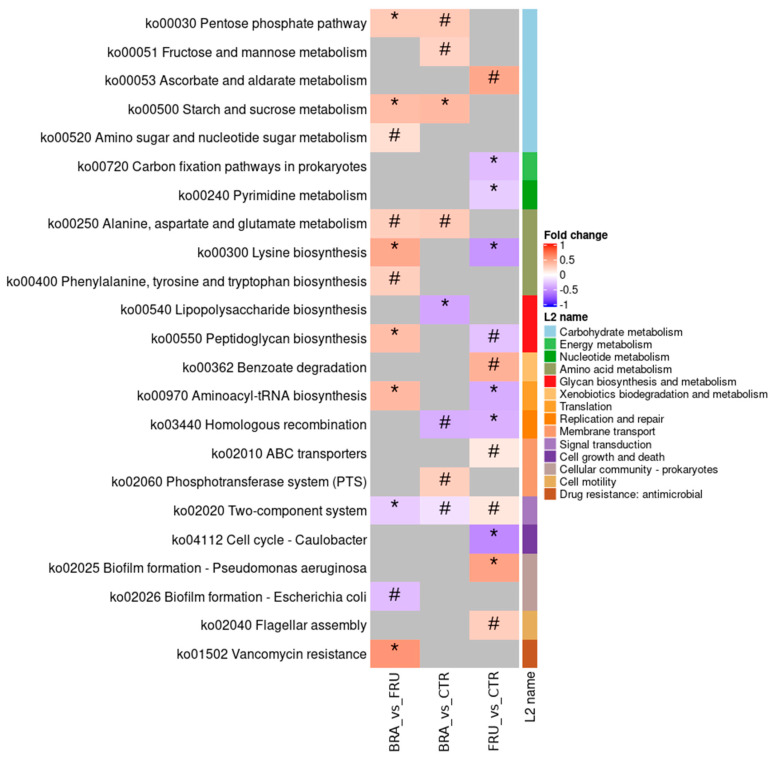
Log 2 fold-change comparison heatmap of the L3 KEGG Orthology terms abundance between treatments. Red means an overabundance of the specific KO term in the former diet of the comparison, and blue means an underabundance. CTR: control diet low in TDF (5.7%); BRA: diet enriched with fiber from cereals (12.0% TDF); FRU: diets enriched with fiber from fruits (12.2% TDF). * *p* < 0.05, # *p* < 0.1.

**Figure A2:**
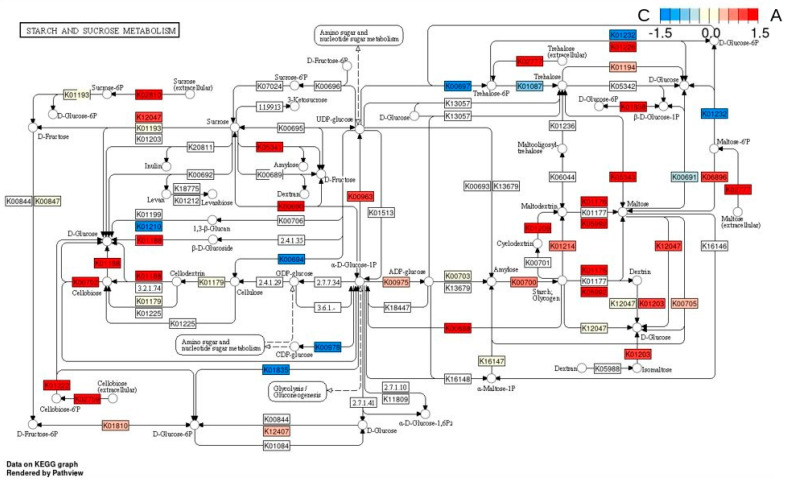
Starch and sucrose metabolic pathway heatmap with log 2 fold-change values of the comparison between CTR (C) and BRA (A) diet. Blue means overabundance of the gene in the CTR group, and red means overabundance in the BRA group. C (CTR)—control diet low in TDF (5.7%); A (BRA)—diet enriched with fiber from cereals (12.0% TDF).

**Figure A3:**
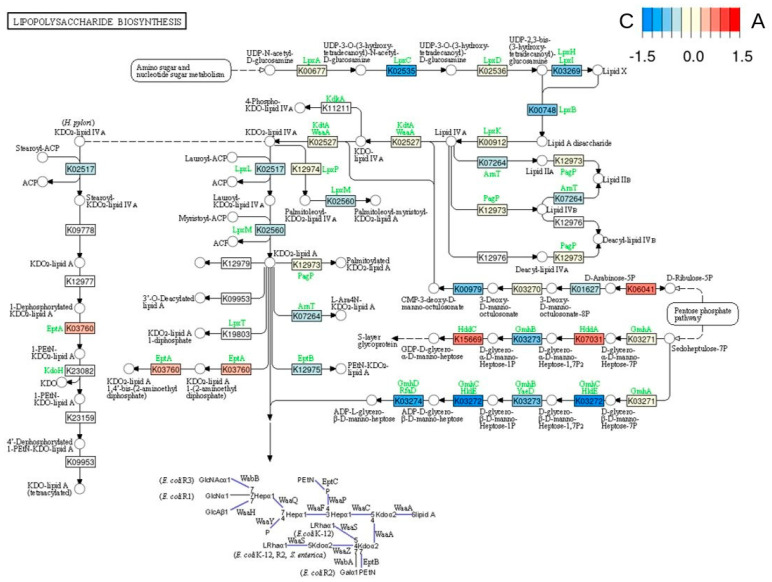
Lipopolysaccharide biosynthesis metabolic pathway heatmap with log 2 fold-change values of the comparison between CTR (C) and BRA (A) diet. Blue means overabundance of the gene in the CTR group, and red means overabundance in the BRA group. C (CTR)—control diet low in TDF (5.7%); A (BRA)—diet enriched with fiber from cereals (12.0% TDF).

**Figure A4:**
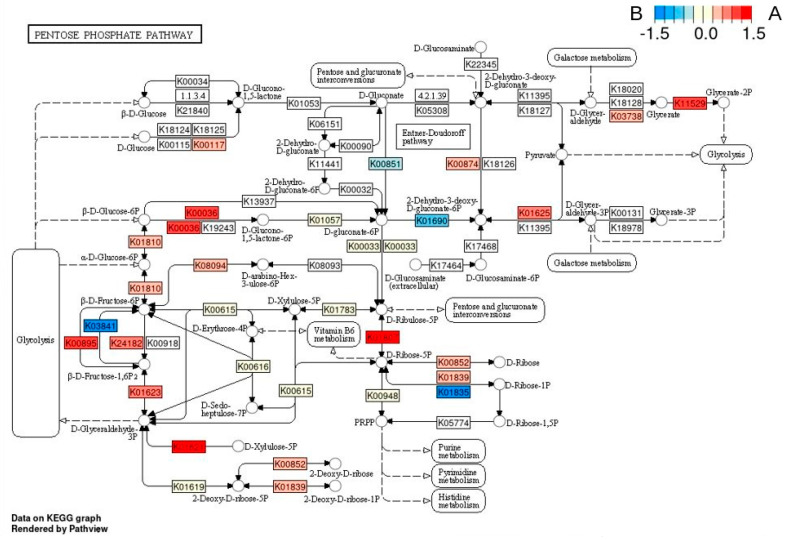
Pentose phosphate metabolic pathway heatmap with log 2 fold-change values of the comparison between FRU (B) and BRA (A) diet. Blue means overabundance of the gene in the FRU group, and red means overabundance in the BRA group. A (BRA)—diet enriched with fiber from cereals (12.0% TDF); B (FRU)—diet enriched with fiber from fruits (12.2% TDF).

For the functional profile analysis of the microbiota, the KEGG database was used. The differential abundance of genes at the L2 level (metabolic pathways) is presented in Figure 5. The BRA diet increased the abundance of starch and sucrose metabolism genes (Figure A2) and decreased the lipopolysaccharide biosynthesis genes (Figure A3) when compared with the CTR diet. Regarding the FRU diet, it decreased cell growth genes, like those related to the cell cycle or tRNA biosynthesis, compared with the CTR. Comparing both fiber-enriched diets, the BRA diet showed a higher abundance of genes related to nutrient metabolism and synthesis, like the pentose phosphate pathway (Figure A4) or lysine synthesis genes.

## 4. Discussion

In the present study, the effects of different combinations of fibrous ingredient sources were investigated. While previous research focused on the impact of isolated or purified sources of fiber in dogs [4,44], very few studies explored the effects of whole ingredients. When a diet is enriched with fibrous ingredients, these ingredients not only provide fiber but also numerous other biologically active compounds with potentially synergistic effects that are challenging to predict. Although in vitro approaches are undeniably useful for such investigations [45], the outcomes in animals do not always reflect these specific predicted effects [46].

In this work, different sources of fiber were used to provide diets with equivalent amounts of soluble and insoluble fiber following a differential strategy. While the BRA diet mostly included fibrous ingredients derived from cereals (oat and wheat), the FRU diet was enriched with fiber from fruits (apple, orange, citrus, and pomegranate). Cereals are known to provide different kinds of fibers, particularly prebiotic compounds, like fructans or β-glucans, which have exhibited functional properties [14,47,48]. On the other hand, fruits are recognized for their pectin contents, which has a prebiotic effect on the host’s microbiota [49]. Additionally, fruits are an excellent source of antioxidants, like polyphenols or flavonoids [50], which are known to have anti-carcinogenic and anti-inflammatory effects [51], along with prebiotic properties [52].

### 4.1. Effects on Fecal Quality and Digestive Function

One of the primary effects of dietary fiber in dog diets is its impact on fecal quality [15,53]. Our study showed that diets enriched with fiber were associated with an increase in fecal volume and water content, even though no differences were found between both enriched diets (Table 3). Typically, insoluble fiber, like cellulose, increases the fecal volume, creating a bulking effect [15], while soluble fiber reduces the dry matter content due to its high water-holding capacity [54]. Therefore, combining soluble and insoluble fiber is often recommended to achieve an appropriate fecal shape, consistency and an optimal fecal score (between 2–3). In our study, we formulated the BRA and FRU diets to have an equivalent ratio of soluble to insoluble fiber, using sugar beet pulp and cellulose. This balanced combination of fibers likely explains the absence of differences in fecal volume or dry matter content between both diets. Remarkably, despite the clear impact of fiber-enriched diets on fecal volume and dry matter, the fecal scores did not differ from the CTR diet, demonstrating that it is possible to increase dietary fiber from 6% to 12% without affecting the fecal quality when the right combination of fibers is used.

Another potential drawback of dietary fiber in dog diets is its potential negative effect on nutrient digestibility. Previous studies reported decreased apparent nutrient digestibility in dogs when dietary fiber is increased [55]. However, if the magnitude of the changes is moderate, the impact on the nutritive value of the diet can be acceptable or negligible in light of the beneficial effects of fiber. In our study, increasing dietary fiber from 6% to 12% resulted in decreases in organic matter and gross energy digestibility of around 5%, around 1–4% for crude protein, and a decrease in fat digestibility, which was observed only with the BRA diet and was around 1% (Table 4). Similar reductions in digestibility due to fiber were reported in dogs by other authors [55,56] and can be attributed to various factors. Insoluble fiber may decrease the gastric transit time and have bulking and laxative effects [57], while soluble fiber could increase the viscosity of the digesta, making it harder for enzymes and substrates to interact [58]. Fiber may also interact with certain lipids and bile acids, reducing their digestibility (e.g., cholesterol) [59] and contributing to the reduction in fat digestion. However, lower digestibility values could also be partially explained by inaccuracies in the apparent digestibility method. The addition of dietary fiber can increase endogenous losses, leading to perceived decreases in energy and nutrient digestibility [60]. In this regard, endogenous components derived from microbial protein can be particularly relevant, as fecal microbial mass can account for undigested dietary protein. In this study, we attempted to correct the apparent crude protein digestibility by subtracting the microbial nitrogen from the feces using purine bases as microbial markers. As a result, differences between the CTR and BRA diet were not significant, and only the FRU diet showed lower protein digestibility (Table 4). In fact, the purine base concentration was higher in the BRA diet than in the FRU diet (Table 3), suggesting a larger microbial population promoted by the former diet.

The apparent digestibility of protein could have also been biased by a variable proteolytic activity of the intestinal microbiota. Proteolytic bacteria could derive part of the undigested crude protein of the diet from feces to the urine in the form of urea. The inclusion of fermentable fiber in the diet was shown to promote the growth of saccharolytic bacterial populations at the expense of proteolytic bacteria [61]. In this regard, our results showed that the fiber-supplemented diets consistently diminished the BCFA concentrations in feces, suggesting a higher supply of fermentable carbohydrates, even at the distal parts of the gut. This reduction in proteolytic activity was particularly evident in the FRU diet, which was associated with lower urea levels in serum. Taken together, it appears that the differences in true protein digestibility were lower than what the apparent digestibility values suggested, and the impact of dietary fiber inclusion on protein digestibility was limited.

### 4.2. Animal Metabolism and Immunity

Notably, the fibrous diets had a significant impact on the serum lipid profile. They tended to reduce the total cholesterol levels, with the BRA diet showing a reduction in serum triglycerides compared with the CTR diet (Table 6). Various mechanisms were proposed to explain these effects [12,59]. For instance, they may inhibit fat hydrolysis by forming gel-like emulsions that prevent lipases from interacting with lipids. Additionally, these diets might downregulate the genes responsible for de novo triglyceride production in the liver. Fermentability appears to be another critical factor that influenced these effects, with propionate potentially exerting an inhibitory effect in the liver. Furthermore, the increased number of bacteria could reduce the availability of cholesterol by capturing it in the membranes of new bacterial cells. The observation that only the BRA diet lowered the serum triglyceride levels could be attributed to the higher fermentability of this diet, as indicated by increased fecal SCFA levels and a larger microbial population, as estimated from the purine base concentrations. Beta-glucans, which are prebiotic compounds found in some cereal crops, such as oats and barley, could also contribute to these effects. Notably, oat beta-glucans were demonstrated to reduce cholesterol and triglyceride concentrations in human subjects [62]. However, it is important to emphasize that while high levels of triglycerides and cholesterol are considered risk factors for metabolic syndrome and cardiovascular diseases in humans, this does not hold the same significance for dogs. Dogs with high cholesterol or other components of metabolic syndrome rarely develop type 2 diabetes, atherosclerosis, coronary heart disease, or stroke [63].

Dietary fiber and prebiotics were demonstrated to potentially have immune-enhancing effects. Supplementation of diets with fiber was shown to modulate the immune response in various areas, including gut-associated lymphoid tissue (GALT), secondary lymphoid tissues, and peripheral circulation [64]. This also applies to dogs, where fiber-rich diets were observed to modulate the type and function of immune cells in the GALT [65]. In our study, the FRU diet appeared to have a mild effect on immunity, leading to an increase in lymphocyte count and percentage, as well as the promotion of CD8+ T lymphocytes (Table 7). However, these effects were not observed with the BRA diet. This differential response could be attributed to the higher levels of polyphenols present in the FRU diet (Table 2). Polyphenols were reported to have immunomodulatory properties [66] and various polyphenol receptors were identified in T cells [67]. This increase in CD8+ T lymphocytes found in the FRU diet could offer numerous health benefits to dogs. First, a decline in this specific lymphocyte type has been associated with both aging and cancer in dogs [68,69]. Moreover, this particular immune cell type demonstrated a connection to heightened resistance against canine visceral leishmaniasis [70]. In summary, these findings underscore the significance of CD8+ lymphocytes in diseases mediated by cellular immunity.

### 4.3. Impact on Intestinal Microbiota: Composition and Functional Changes

In the present study, we employed several methodologies to gain insights into the potential impacts of the diets on microbiota composition and activity. We assessed the SCFA production and conducted metagenome sequencing to evaluate the microbiota functionality, while 16S rRNA sequencing was used to determine the microbiota taxonomical distribution. The microbiota’s composition was dominated by the *Bacteroidetes*, *Firmicutes*, and *Fusobacteria* phyla, with *Bacteroidetes* being the most abundant (Figure A1). Although our study showed *Bacteroidetes* as the dominant phylum, it is worth noting that *Firmicutes* typically represent this group in dogs [71,72]. Despite this variation, these results align with the most commonly abundant phyla in dogs [73].

Increased dietary fiber intake with the experimental diets had a clear impact on fermentation (Table 5). Both diets led to increased fecal SCFA concentrations (*p* = 0.069), which is consistent with findings by other authors [23,74]. These higher concentrations of SCFAs translated into higher concentrations of acetate and, particularly, butyrate (*p* = 0.074), which offer potential benefits for the host. Butyrate, in particular, is the preferred energy source for the colonocyte and possesses anti-inflammatory, antioxidant, and anticarcinogenic properties [75]. Butyrate has also been described to promote lipogenesis and the synthesis of many key components for the intestinal epithelium, increasing epithelial cell proliferation and differentiation and improving colonic barrier function [76].

The observed increase in butyrate concentration with fibrous diets was primarily due to increased fermentative activity, rather than changes in specific butyrogenic bacteria. Notably, when we analyzed the SCFAs in terms of molar proportions, fibrous diets induced a more acetogenic and less butyrogenic fermentation than the control (CTR) diet (Table 5). Similar shifts in the fermentation profile were observed by other researchers who supplemented dog diets with citrus pulp or orange fiber [23]. In an attempt to identify potential butyrogenic species, we conducted correlations between SCFAs and specific amplicon sequence variants (ASVs) (Figure 4). Surprisingly, we found that six different ASVs, identified as *Alloprevotella*, were negatively correlated with fecal butyrate levels. *Alloprevotella* is typically identified as a fiber fermenter and butyric acid producer [77,78], suggesting that this negative correlation could be attributed to changes in particular non-butyrogenic species within this genus. However, these taxa did not appear to be significantly affected by the diet.

As for changes in BCFAs resulting from the fermentation of branched amino acids, both the BRA and FRU diets were associated with decreases in fecal BCFA concentrations and molar proportions (Table 5). This confirmed the relevant impacts of these diets in reducing the proteolytic activity of the microbiota by guaranteeing the supply of fermentable carbohydrates throughout the gastrointestinal tract. This reduction in proteolytic activity can be considered beneficial, as protein putrefaction can generate compounds such as ammonia, amines, phenol, indole, and sulfides, all of which have been associated with negative effects on animal health [79]. Additionally, specific ASVs, such as *Phascolarctobacterium succinatutens*, showed a positive and significant correlation with BCFAs, with this species being less abundant in the fiber-supplemented diet groups (Figure 4). These results suggest the potential of *Phascolarctobacterium succinatutens* to serve as a taxonomic biomarker.

Regarding the distinct effects of the BRA diet, it notably increased the alpha diversity compared with the FRU diet (Figure 1). This outcome implies that cereal fiber sources have a greater capacity to increase microbiota biodiversity, enhancing its robustness and resilience [80]. The ability of whole cereals, particularly bran-derived fractions to prevent disease in humans and promote health by modulating gut microbiota, were described in a multitude of studies [81,82]. The high content of hemicelluloses and arabinoxylans found in cereals and its high structural heterogeneity [83] could be the reason behind this higher impact on ecosystem complexity. Sugar beet pulp also added various fermentative compounds to the mixture, such as pectins, which may also contribute to the observed effects [84]. Additionally, the BRA diet appeared to have a stronger prebiotic effect compared with the FRU diet (Figure 3). It promoted the growth of beneficial bacterial genera, such as *Bifidobacterium*, which are generally considered beneficial in humans [85], though they may play a less important role in dogs [86]. The BRA diet also fostered the growth of fiber-degrading and SCFA-producing bacteria, like *Fecalibacterium*, which is a butyrate producer [87], and *Lachnospira*, both of which have been associated with healthy dogs fed high-fiber diets [88,89]. In addition to the impact on microbiota composition, the BRA diet exhibited an overabundance of KO term genes related to starch and sucrose metabolism (Figure 5). This could be attributed to its enhanced capacity for degrading fibrous compounds. Lin et al. [71] also reported an overexpression of genes associated with carbohydrate metabolism in dogs fed a high-fiber diet based on corn fiber. Moreover, the BRA diet, in comparison with the FRU diet, showed an overabundance of genes related to amino acid metabolism and glycan biosynthesis. This could suggest a higher metabolic activity and an increase in bacterial growth. In fact, the BRA diet was associated with higher levels of fecal acetate and purine bases, which might be indicative of a more active microbial population of larger size.

On the other hand, the FRU diet led to specific changes in certain microbial groups. Similar to the BRA diet, it increased the presence of fiber-fermenting bacteria, like *Fecalibacterium* and *Phocaeicola* [90], albeit to a lesser extent (Figure 3). The FRU diet also promoted an increase in the *Selenomonadaceae* family (Table A2), which was observed to increase in dogs administered kefir, which is a probiotic dairy product [91]. This family of bacteria was negatively correlated with aggression scores and positively correlated with body condition scores in dogs [92]. Notably, the FRU diet resulted in significant reductions in several microbial groups, particularly some potentially harmful genera. These reductions encompassed genera such as *Erysipelatoclostridium* (Table 3), which is known to cause invasive infections in immunocompromised humans [93], to be associated with obesity in mice [94], and to be increased in parvovirus-infected dogs [95]. Additionally, other genera, like *Lacrimispora*, were diminished in the FRU diet. A study involving human milk oligosaccharides supplementation in humans also reported a decrease in *Lacrimispora* within the supplemented group [96]. *Oribacterium*, which is another bacterial genus, declined with the administration of the FRU diet and has been associated with a normal body condition score in dogs [91].

These reductions in specific microbial groups observed with the FRU diet might be attributed to the selective suppression of certain bacterial taxa due to its higher content of polyphenols. Some polyphenols are known to possess antibacterial activity and the ability to modulate the gut microbiota [97,98]. This selective antibacterial activity could also explain the lower richness observed in the FRU diet, especially when compared with the BRA diet. Moreover, the higher polyphenol content in the FRU diet might also be responsible for the observed effects on microbiota functionality, including the underabundance of genes related to the cell cycle, tRNA biosynthesis, replication, and repair.

Fruits sources included in the FRU diet, apple, orange, and pomegranate, are known to be rich in different polyphenolic compounds [99]. In fact, the FRU diet exhibited higher levels of total polyphenols compared with the other two diets (see Table 2; 2500 vs. 1500 mg/kg). Polyphenols are resistant to digestion and reach the hindgut, where they can be metabolized into secondary bioactive compounds by the microbiota [100]. These metabolites are considered to be the most bioactive forms of polyphenols [101]. It can be hypothesized that the increase in these bioactive compounds promoted by the FRU diet may also account for the observed effects on the immune response, as indicated by higher levels of total and CD8 lymphocytes.

## 5. Conclusions

The selection of fiber sources significantly influences host response and gut microbiota. In this study, diets enriched with cereal-based fiber sources led to a more diverse gut ecosystem, resulting in increased alpha diversity and a larger fecal microbial population compared with fruit-based diets. Additionally, cereal fiber and beet pulp content exhibited a stronger prebiotic effect by fostering the growth of beneficial genera, like *Bifidobacterium* and butyrate-producing *Fecalibacterium*, along with an overabundance of genes related to carbohydrate and amino acid metabolism. Cereal-based sources also affected lipid metabolism, resulting in reduced blood triglycerides. Conversely, diets with fruit-based sources had distinct impacts on bacterial taxonomy and microbiota functionality compared with cereals and led to lower cholesterol levels in animals. These effects may be attributed to the higher polyphenol content in fruits, potentially driving changes in the gut ecosystem and impacting the animals’ immune response, as suggested by elevated levels of total and CD8 lymphocytes. Further studies are necessary to explore the specific effects of individual fibrous ingredients and the potential influence of fruit polyphenols on gut microbiota and animal health.

## Figures and Tables

**Table 1:** Differential fibrous ingredients included in each of the experimental diets (% as fed).

BRA	FRU
Wheat midds (10%)	Citrus pulp (5%)
Oat bran (6%)	Apple fiber (3.5%)
Oat fiber (1.5%)	Orange peel (0.6%)
Sugar beet pulp (5.5%)	Pomegranate peel (0.5%)
	Cellulose pellets (2.5%)

Notes: Sugar beet pulp and cellulose pellets were used as sources of soluble and insoluble fiber to formulate diets for an equivalent insoluble/soluble ratio. Additionally, fructo-oligosaccharides (FOS), as a purified source of soluble fiber, were included in both diets at the same level (0.5%).

**Table 2:** Analyzed chemical compositions of experimental diets (as fed).

	CTR	BRA	FRU
Dry matter (%)	92.6	92.3	92.7
Crude protein (%)	27.0	26.1	25.5
Crude fat (%)	15.1	15.7	15.6
Ashes (%)	7.2	6.8	6.5
NDF (%)	9.5	14.0	13.0
ADF (%)	2.1	4.2	5.4
ADL (%)	1.0	1.4	1.8
Total dietary fiber (%)	5.7	12.0	12.2
Soluble dietary fiber (%)	1.4	1.1	1.3
Insoluble dietary fiber (%)	5.8	10.3	10.6
Total polyphenols (mg/kg)	1500	1500	2500
Gross energy (kcal/kg)	4777	4776	4794

Notes: CTR—control diet low in fiber; BRA—diet with supplemented fiber coming from cereals; FRU—diet with supplemented fiber coming from fruits; NDF—neutral detergent fiber; ADF—acid detergent fiber; ADL—acid detergent lignin.

**Table 3:** Fecal quality of dogs (*n* = 12) fed extruded diets, including different levels and sources of total dietary fiber (TDF).

	CTR	BRA	FRU	*p*-Value
Fecal score	2.81 ± 0.419	2.67 ± 0.324	2.62 ± 0.386	0.167
Fecal dry matter (%)	35.2 ^a^ ± 2.30	29.2 ^b^ ± 1.50	29.1 ^b^ ± 1.95	<0.001
Fecal mass (g FM/day)	82.0 ^a^ ± 15.98	147.7 ^b^ ± 32.75	152.5 ^b^ ± 29.75	<0.001
Fecal bacterial N (mg/g DM)	50.5 ^ab^ ± 17.68	58.8 ^a^ ± 20.00	44.2 ^b^ ± 19.86	0.043

Note: CTR—control diet low in TDF (5.7%); BRA—diet enriched with fiber from cereals (12.0% TDF); FRU—diets enriched with fiber from fruits (12.2% TDF). All were extruded diets. FM—fresh matter; DM—dry matter. Means within a row with different superscripts showed statistical differences (*p* < 0.05).

**Table 4:** Apparent nutrient digestibility values and estimated metabolizable energy of the experimental diets tested on dogs (*n* = 12). Data represented as means and standard deviations.

	CTR	BRA	FRU	*p*-Value
Dry matter (%)	87.7 ^a^ ± 2.60	82.8 ^b^ ± 4.12	82.5 ^b^ ± 4.38	<0.001
Organic matter (%)	91.5 ^a^ ± 1.74	86.0 ^b^ ± 3.46	85.1 ^b^ ± 3.79	<0.001
Crude protein (%)	87.8 ^a^ ± 2.68	85.2 ^b^ ± 3.64	83.1 ^c^ ± 4.00	<0.001
Corrected crude protein (%)	89.9 ^a^ ± 2.65	88.8 ^a^ ± 3.15	85.8 ^b^ ± 4.02	<0.001
Crude fat (%)	96.6 ^a^ ± 0.62	95.3 ^b^ ± 1.02	96.3 ^a^ ± 1.01	<0.001
Hemicellulose (%)	80.0 ^a^ ± 5.45	74.9 ^b^ ± 6.23	76.0 ^b^ ± 6.20	0.005
Cellulose (%)	34.7 ^a^ ± 14.29	32.9 ^a^ ± 14.49	20.8 ^b^ ± 19.37	0.001
Gross energy (%)	91.6 ^a^ ± 1.73	86.7 ^b^ ± 3.22	86.2 ^b^ ± 3.40	<0.001
Metabolizable energy (kcal/kg as fed), FEDIAF	4058 ^a^ ± 73	3843 ^b^ ± 141	3846 ^b^ ± 150	<0.001

Note: CTR—control diet low in TDF (5.7%); BRA—diet enriched with fiber from cereals (12.0% TDF); FRU—diets enriched with fiber from fruits (12.2% TDF). All were extruded diets. FEDIAF—Fédération Européenne De l’Industrie des Aliments pour Animaux Familiers. Means within a row with different superscripts showed statistical differences (*p* < 0.05).

**Table 5:** Short-chain and branched-chain fatty acid concentrations in fresh dog feces (*n* = 12) and their respective molar ratio percentages of total SCFAs. Data represented as means and standard deviations.

Concentration (µmol/g)	CTR	BRA	FRU	*p*-Value
Total SCFAs	138.1 ± 41.04	177.1 ± 42.87	160.3 ± 44.10	0.069
Acetate	75.8 ^a^ ± 20.29	102.7 ^b^ ± 23.42	92.0 ^ab^ ± 23.54	0.023
Propionate	39.3 ± 12.07	48.4 ± 14.86	41.8 ± 11.69	0.178
Butyrate	15.7 ± 7.56	19.9 ± 6.86	21.2 ± 9.97	0.074
Valerate	1.36 ± 1.420	1.75 ± 2.375	0.87 ± 0.748	0.498
Total BCFAs	6.01 ^a^ ± 1.602	4.35 ^b^ ± 0.868	4.45 ^b^ ± 0.881	0.001
Molar ratio (%SCFAs)				
Acetate	55.2 ^a^ ± 2.60	58.5 ^b^ ± 5.06	57.7 ^b^ ± 2.53	0.004
Propionate	28.5 ± 1.92	26.9 ± 3.28	26.1 ± 1.22	0.054
Butyrate	2.35 ^a^ ± 0.320	1.43 ^b^ ± 0.260	1.67 ^b^ ± 0.599	<0.001
Valerate	0.94 ± 0.891	1.05 ± 1.401	0.56 ± 0.478	0.540
Total BCFAs	4.39 ^a^ ± 0.587	2.50 ^b^ ± 0.335	2.94 ^b^ ± 0.845	<0.001

Note: CTR—control diet low in TDF (5.7%); BRA—diet enriched with fiber from cereals (12.0% TDF); FRU—diets enriched with fiber from fruits (12.2% TDF). All were extruded diets. SCFAs—short-chain fatty acids; BCFAs—branched-chain fatty acids. Means within a row with different superscripts showed statistical differences (*p* < 0.05).

**Table 6:** Lipid profile and glucose and urea concentrations in serum of dogs (*n* = 12) fed different diets with different levels and sources of dietary fiber. Data represented as means and standard deviations.

	CTR	BRA	FRU	*p*-Value
Total cholesterol (mg/dL)	214.7 ± 28.04	207.0 ± 22.89	200.8 ± 26.99	0.090
HDL cholesterol (mmol/L)	4.26 ± 0.524	4.21 ± 0.414	4.16 ± 0.668	0.731
LDL cholesterol (mmol/L)	1.55 ± 0.397	1.51 ± 0.290	1.48 ± 0.163	0.507
Triglycerides (mg/dL)	44.8 ^a^ ± 14.21	32.8 ^b^ ± 4.26	36.9 ^ab^ ± 10.01	0.007
Glucose (mg/dL)	108.0 ± 6.17	108.1 ± 7.40	110.9 ± 5.45	0.081
Urea (mg/dL)	30.7 ^a^ ± 7.08	29.8 ^ab^ ± 6.70	26.9 ^b^ ± 3.42	0.010

Note: CTR—control diet low in TDF (5.7%); BRA—diet enriched with fiber from cereals (12.0% TDF); FRU—diets enriched with fiber from fruits (12.2% TDF). All were extruded diets. Means within a row with different superscripts showed statistical differences (*p* < 0.05).

**Table 7:** Complete blood cell and lymphocyte subset counts of dogs (*n* = 12) fed different diets with different levels and sources of dietary fiber. Data represented as means and standard deviations.

	CTR	BRA	FRU	*p*-Value
Leucocytes (count^3^/μL)	7.21 ± 1.472	7.75 ± 1.805	7.56 ± 2.355	0.7471
Lymphocytes (%leucocytes)	29.1 ^ab^ ± 6.51	26.7 ^a^ ± 8.00	31.0 ^b^ ± 8.05	0.0390
Monocytes (%leucocytes)	7.89 ± 1.185	7.28 ± 1.760	7.91 ± 1.230	0.4037
Neutrophils (%leucocytes)	58.9 ± 6.52	62.7 ± 9.89	57.8 ± 8.31	0.0598
CD8 (count^3^/μL)	0.48 ^ab^ ± 0.268	0.42 ^a^ ± 0.200	0.50 ^b^ ± 0.263	0.0407
CD4 (count^3^/μL)	0.75 ± 0.321	0.70 ± 0.244	0.80 ± 0.304	0.2551
Ratio CD4/CD8	1.86 ± 0.872	1.90 ± 0.775	1.82 ± 0.714	0.7702

Note: CTR—control diet low in TDF (5.7%); BRA—diet enriched with fiber from cereals (12.0% TDF); FRU—diets enriched with fiber from fruits (12.2% TDF). All were extruded diets. Means within a row with different superscripts showed statistical differences (*p* < 0.05).

## Data Availability

The data presented in this study are available on request from the corresponding author. The data are not publicly available due to privacy restrictions.

## Appendix A

**Table A1:** Ingredient list of the control (CTR) diet, to which the supplemental fiber was added to produce BRA and FRU diets.

CTR Ingredient List
Chicken, corn, rice, poultry, palatants, potato protein, fish oil, minerals, and vitamins

**Table A2:** Twenty-five most abundant families in dog’s fecal microbiota by relative abundance represented in percentage with its standard deviation.

	CTR	BRA	FRU
*Prevotellaceae*	26.44 ± 5.312	24.07 ± 5.581	21.72 ± 6.071
*Fusobacteriaceae*	20.38 ± 7.235	16.26 ± 7.968	20.45 ± 10.793
*Bacteroidaceae*	15.25 ± 5.198	14.64 ± 5.731	13.90 ± 6.038
*Sutterellaceae*	7.27 ± 3.700	9.48 ± 4.632	9.52 ± 5.148
*Muribaculaceae*	4.10 ± 4.903	7.33 ± 5.300	5.29 ± 6.240
*Acidaminococcaceae*	6.74 ± 1.881	4.70 ± 2.269	4.06 ± 2.220
*Lachnospiraceae*	3.87 ± 2.631	4.70 ± 2.389	3.90 ± 3.912
*Lactobacillaceae*	3.74 ± 5.299	3.56 ± 3.346	4.64 ± 5.665
*Oscillospiraceae*	1.97 ± 1.826	3.79 ± 2.489	2.17 ± 1.285
*Succinivibrionaceae*	2.59 ± 2.717	2.04 ± 1.735	2.96 ± 2.217
*Selenomonadaceae*	0.73 ± 0.778 ^a^	1.18 ± 1.524 ^ab^	5.08 ± 6.520 ^b^
*Erysipelotrichaceae*	2.26 ± 2.382	2.41 ± 1.657	1.51 ± 1.119
*Turicibacteraceae*	0.70 ± 0.806	1.35 ± 2.569	2.03 ± 3.071
*Peptostreptococcaceae*	1.20 ± 0.709	1.71 ± 2.208	0.85 ± 0.413
*Tannerellaceae*	0.53 ± 0.237	0.38 ± 0.251	0.27 ± 0.228
*Coriobacteriaceae*	0.60 ± 0.741	0.28 ± 0.163	0.29 ± 0.202
*Rikenellaceae*	0.46 ± 0.494	0.33 ± 0.277	0.25 ± 0.330
*Clostridiaceae*	0.18 ± 0.141	0.53 ± 0.420	0.33 ± 0.289
*Coprobacillaceae*	0.07 ± 0.132	0.23 ± 0.509	0.24 ± 0.490
*Eubacteriales* Family XIII. *Incertae Sedis*	0.11 ± 0.068	0.18 ± 0.209	0.06 ± 0.082
*Bifidobacteriaceae*	0.02 ± 0.045 ^a^	0.22 ± 0.233 ^b^	0.06 ± 0.117 ^ab^
*Enterobacteriaceae*	0.07 ± 0.222	0.17 ± 0.481	0.05 ± 0.097
*Eggerthellaceae*	0.09 ± 0.160	0.10 ± 0.094	0.06 ± 0.059
*Eubacteriales* noname	0.13 ± 0.264	0.05 ± 0.045	0.03 ± 0.039
*Deferribacteraceae*	0.03 ± 0.065	0.02 ± 0.042	0.04 ± 0.050

Note: CTR—control diet low in TDF (5.7%); BRA—diet enriched with fiber from cereals (12.0% TDF); FRU—diet enriched with fiber from fruits (12.2% TDF). Mean values with different superscripts in the same row differ with *p* < 0.05.
